# Supplementary material for: Characterization of the Outer Membrane Vesicles of Pseudomonas aeruginosa Exhibiting Growth Inhibition against Acinetobacter baumannii
Source: Biomedicines. 2024 Mar 1;12(3):556. doi: 10.3390/biomedicines12030556 (PMC10967770; doi:10.3390/biomedicines12030556)
Supplement: Supplementary file 1 [file biomedicines-12-00556-s001.zip › biomedicines-2871992-supplementary.pdf]

Supplementary Materials

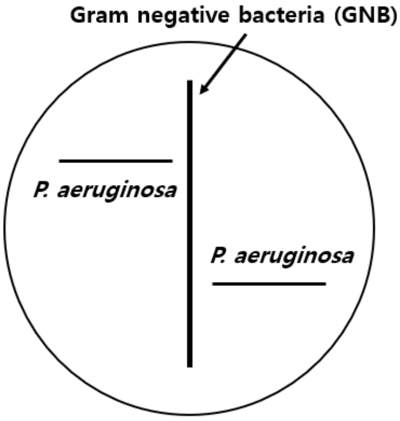

Figure S1. Illustration of the design of the modified cross-streak assay. GNB included *A. baumannii.*

Table S1. Characteristics of gram-negative bacteria in clinical samples

Strains	Sample	Ampicillin	Cefotaxime	Cefepime	Ciprofloxacin	Gentamicin	Amikacin	Imipenem	PIP/TAZ	TMP/SMX
Pseudomonas aeruginosa										
ATCC 27853	blood		R 8	S ≤ 1	S ≤ 0.5	S ≤ 1	S ≤ 2	S 2	S ≤ 4	R ≥ 320
PA 022	urine	R ≥ 32	R 8	S 4	S 1	S ≤ 1	S ≤ 2	S 1	S 8	R ≥ 320
PA 068	blood	R ≥ 32	R 8	S 8	I 2	S ≤ 1	S ≤ 2	R ≥ 16	S 8	R 160
PA 008	sputum	R ≥ 32	S 4	R>16	R>2	R>8	R>32	R>8	R>64	R ≥ 320
		**AMP/SUL**	**Cefotaxime**	**Cefepime**	**Ciprofloxacin**	**Gentamicin**	**Amikacin**	**Imipenem**	**PIP/TAZ**	**TMP/SMX**
Acinetobacter baumannii										
AB MIC34	environment	R ≥ 32	R ≥ 64	R ≥ 64	R ≥ 4	R ≥ 16	R ≥ 16	R ≥ 16	R ≥ 128	R ≥ 320
AB 004	blood	S ≤ 2	S 8	S 2	I 2	S ≤ 1	S ≤ 2	S ≤ 0.25	S ≤ 4	S ≤ 20
AB 037	blood	R ≥ 32	R ≥ 64	R ≥ 64	R ≥ 4	R ≥ 16	S 8	R ≥ 16	R ≥ 128	R 80
AB SSH20	blood	I 16	R ≥ 64	R ≥ 64	R ≥ 4	R ≥ 16	R ≥ 64	R ≥ 16	R ≥ 128	R ≥ 320
AB KBU02	blood	R ≥ 32	R ≥ 64	R 32	R ≥ 4	R ≥ 16	I 32	R ≥ 16	R ≥ 128	R ≥ 320

Abbreviation: AMP/SUL, ampicillin-sulbactam; PIP/TAZ, piperacillin-tazobactam; TMP/SMX, trimethoprim-sulfamethoxazol

Table S2. Outer membrane and extracellular proteins identified in *P. aeruginosa* OMVs (PA ATCC 27853)

Protein name (PA ATCC 27853)	No.	Molecular function	Average Mass (kDa)	Gene Name	Subcellular localization
Uncharacterized protein	27				unknown
Putative permease	2	transmembrane transport	39217.00	yjgQ	cytoplasmic membrane
Probable queuosine precursor transporter	13	queuosine salvage<br>transport	24344.92	yhhQ	cytoplasmic membrane
MFS transporter (Inner membrane transport protein ydhP)	27		34248.35	ydhP_2	cytoplasmic membrane
Outer membrane protein assembly complex YaeT protein	2	Gram-negative-bacterium-type cell outer membrane assembly<br>protein insertion into membrane	87678.00	yaeT	outer membrane
Protein tolQ	13	bacteriocin transport<br>cell cycle<br>cell division	23713.32	tolQ	cytoplasmic membrane
Protein-export membrane protein SecG	14	protein secretion	13210.00	secG	cytoplasmic membrane
Succinate dehydrogenase cytochrome b556 subunit	12	tricarboxylic acid cycle	15415.13	sdhC	cytoplasmic membrane
50S ribosomal protein L4	15	translation	21074.13	rplD	cytoplasmic
MFS family transporter	3		39914.00	Q058_05356	cytoplasmic membrane
Membrane protein putative	2	transmembrane transport	47933.00	PSPA7_1284	cytoplasmic membrane
Cytochrome b	22	respiratory electron transport chain<br>Electron Transport<br>Respiratory chain<br>Transport	46111.25	petB	cytoplasmic membrane
Peptidoglycan-associated protein	14	cell cycle<br>cell division	17963.58	pal	outer membrane
Lipid A deacylase	4		18399.60	pagL	outer membrane
Lipid A 3-O-deacylase	3		18394.00	pagL	outer membrane
Multidrug efflux RND transporter outer membrane subunit OprN	24		51163.11	oprN	outer membrane
Peptidoglycan-associated lipoprotein OprL.	7		16841.40	oprL	outer membrane
Multidrug efflux RND transporter permease subunit MexF	17	xenobiotic transport	115463.20	mexF	cytoplasmic membrane
LPS export ABC transporter permease LptG	22	transmembrane transport	39192.36	lptG	cytoplasmic membrane
Signal peptidase I	23	signal peptide procssing	31981.87	lepB	cytoplasmic mem

					brane
7-cyano-7-deazaguanine/7-aminomethyl-7-deazaguanine transporter	8	queuosine salvage	24222.75	IPC574_26295	cytoplasmic membrane
Transporter	3		45130.33	IPC1518_14570	outer membrane
Clp protease ClpP	5	proteolysis	73919.40	IPC1517_20515	cytoplasmic
Cytochrome bc complex cytochrome b subunit	9	respiratory electron transport chain	46041.11	IPC137_16875	cytoplasmic membrane
Glutamate/aspartate:proton symporter GltP	23	carboxylic acid transport	46658.43	gltP	cytoplasmic membrane
Long-chain fatty acid transport protein	3		45566.67	fadL1	outer membrane
ATP-dependent Clp protease proteolytic subunit	2	proteolysis	73683.00	CAZ10_31510	cytoplasmic
Putative membrain protein	2		56813.00	BN889_01892	cytoplasmic membrane
Outer membrane protein assembly factor BamA	11	Gram-negative-bacterium-type cell outer membrane assembly<br>protein insertion into membrane	88435.73	bamA	outer membrane
AmpDh3	4	peptidoglycan catabolic process<br>peptidoglycan turnover<br>regulation of beta-lactamase activity	28739.33	ampDh3	cytoplasmic
N-acetylmuramoyl-L-alanine amidase	22	peptidoglycan catabolic process	28721.14	amiD_1	cytoplasmic
N-acetylmuramoyl-L-alanine amidase domain-containing protein	3	peptidoglycan catabolic process	28240.67	ALP65_01634	cytoplasmic
Succinate dehydrogenase C subunit	1		13771.00	sdhC	cytoplasmic membrane
Proton glutamate symporter protein	1		47621.00	Q041_06569	cytoplasmic membrane
LPS export ABC transporter permease LptF	1		41343.00	lptF	cytoplasmic membrane
Inactive transglutaminase family protein	3		56812.00	J9247_12845	cytoplasmic membrane
Phage tail protein	2		72190.00	IPC65_03235	extracellular
TolC family protein	1		47611.00	IPC183_04225	outer membrane
Bacteriophage protein	1		17953.00	IPC1316_15330	unknown
Efflux transporter outer membrane factor (OMF) lipo NodT family protein	1		51290.00	CSB93_4029	outer membrane

RND transporter	1		51161.00	CAZ10_13505	outer membrane
Outer membrane protein transport protein	3		45562.00	ALP65_01642	outer membrane
Porin D	1	transmembrane transport	46900	oprD3	outer membrane

Table S3. Outer membrane and extracellular proteins identified in *P. aeruginosa* OMVs (PA 022)

Protein name (PA 022)	No.	Molecular function	Average Mass	Gene Name	Subcellular localization
Uncharacterized protein	65				unknown
Sec translocon accessory complex subunit YajC	31	protein transport	11800.79	yajC	cytoplasmic membrane
Soluble pyridine nucleotide transhydrogenase	17	Conversion of NADPH, generated by peripheral catabolic pathways, to NADH, which can enter the respiratory chain for energy generation.	51228.47	sthA	cytoplasmic
Glycine zipper 2TM domain-containing protein	7	unreviewed	15660.67	slyB_1	outer membrane
30S ribosomal protein S20	15	Binds directly to 16S ribosomal RNA.	9911.00	rpsT	cytoplasmic
Secreted lipoprotein	2	unreviewed	20620.00	Q058_05429	periplasmic
Rick_17kDa_Anti domain-containing protein	5	unreviewed	15649.00	Q058_03352	outer membrane
TonB-dependent copper receptor	26	receptor	79249.31	Q058_00426	outer membrane
Outer membrane efflux protein	4	efflux transmembrane transporter activity	53298.00	PSPA7_5705	outer membrane
Lipoprotein putative	5	unreviewed	14798.00	PSPA7_4325	outer membrane
Phospholipase	18	phospholipase C activity, zinc ion binding	36729.43	plcB	unknown
Pilin	3	cell adhesion, protein secretion by the type II secretion system	15958.33	pilA	extracellular
Ubiquinol-cytochrome c reductase iron-sulfur subunit	12	ubiquinol-cytochrome-c reductase activity	20592.18	petA	cytoplasmic membrane
Lipid A deacylase	7	Has lipid A 3-O-deacylase activity. Hydrolyzes the ester bond at the 3 position of lipid A, a bioactive component of lipopolysaccharide (LPS), thereby releasing the primary fatty acyl moiety.	18403.86	pagL	outer membrane
Lipid A 3-O-deacylase	2	structural constituent of ribosome	18394.00	pagL	cytoplasmic
Putative secreted lipoprotein	2	unreviewed	20620.00	PA14_57510	periplasmic
Probable bacteriophage protein	2	unreviewed	29574.50	PA0622	unknown
Bacteriophage protein	2	unreviewed	29574.50	PA0622	unknown
Outer membrane protein OprM	28	efflux transmembrane transporter activity, response to antibiotic	52607.55	oprM	outer membrane
Outer membrane protein oprJ	12	efflux transmembrane transporter activity	51955.83	oprJ	outer membrane
Outer membrane porin protein OprD	10	unreviewed	47550.67	OprD	outer membrane
Copper transport outer membrane porin OprC	5	unreviewed	78644.00	oprC	outer membrane
Channel protein TolC	21	efflux transmembrane transporter activity	53345.61	opmH	outer membrane
Neutral metalloproteinase (Elastase, virulence factor)	19	Extracellular zinc metalloprotease.	51771.00	lasB	extracellular
Si-specific NAD(P)(+) transhydrogenase	11	Conversion of NADPH, generated by peripheral catabolic pathways, to NADH, which can enter the respiratory chain for energy generation.	51231.36	IPC1590_16135	cytoplasmic

Tail length tape measure protein	5	unreviewed	114358.60	IPC1499_31525	unknown (multiple localization sites)
Ig-like domain-containing protein	9	unreviewed	41001.89	IPC1476_20335	unknown
YgdI/YgdR family lipoprotein	2	Has lipid A 3-O-deacylase activity. Hydrolyzes the ester bond at the 3 position of lipid A, a bioactive component of lipopolysaccharide (LPS), thereby releasing the primary fatty acyl moiety.	8095.00	IPC1338_27820	outer membrane
BON domain-containing protein	13	unreviewed	20658.50	IPC1337_16380	periplasmic
Ig domain-containing protein	3	unreviewed	40971.67	IPC1295_32675	unknown
Peptidase M4 family protein	10	Extracellular zinc metalloprotease	53672.10	IPC113_02740	extracellular
Phage tail protein	17	the pyocin r2 phage p2 tail fiber protein (pyocin r2) family	68141.85	HR1 (Hypervariable region 1)	extracellular
Flagellin	30	Structural molecular activity	31929.25	fliC	extracellular
Lipoprotein	2		8109.00	CSB93_4084	unknown
Phage major tail tube protein	4	unreviewed	17914.25	CAZ10_19115	unknown
Putative bacteriophage protein	2	unreviewed	45473.00	BN889_00727	unknown
ATP synthase subunit c	22	F_1F_0 ATP synthase produces ATP from ADP in the presence of a proton or sodium gradient.	8636.41	atpE	cytoplasmic membrane
Chromosome partition protein Smc	1		114403.00	smc	unknown (multiple localization sites)
17 kDa surface antigen	1		15649.00	slyB	outer membrane
Pseudolysin	1		53687.00	Q041_00565	extracellular
Putative outer membrane protein	1		46879.00	PSPA7_2476	outer membrane
Secreted protein	1		59984.00	PA52Ts2_0073	unknown
YadA-like family protein	1		90268.00	K3T18_31340	outer membrane
DUF2184 domain-containing protein	1		33741.00	IPC1485_10470	unknown
FIIR2 protein	1		18071.00	FIIR2	unknown
OOP family OmpA-OmpF porin	1	lipid binding	37360.00	DFO60_2275 (atpE)	cytoplasmic membrane
Phospholipid-binding protein	1		20728.00	CEG18_25265	periplasmic
F0F1 ATP synthase subunit C	1		8608.00	atpE	unknown
Pyr_redox_2 domain-containing protein	1	oxidoreductase activity	39630.00	ALP65_00821	cytoplasmic

Table S4. Outer membrane and extracellular proteins identified in *P. aeruginosa* OMVs (both PA 022 and PA ATCC 278953)

Protein name	No.	Molecular function	Average Mass	Gene Name	Subcellular localization
Uncharacterized protein	8				
Phage tail sheath protein	1		41210.00	Q058_05229	unknown
Acyloxyacyl hydrolase	11		18543.63	IPC1311_20620	unknown
Phage tail sheath family protein	3		41167.00	DT376_09980	unknown
Outer membrane OprD family porin	2		46851.00	Q041_01596	outer membrane
Probable outer membrane protein	2	porin activity	46206.50	PA2760	outer membrane
OprE3	1	membrane	46837.00	oprQ	outer membrane
Outer membrane lipoprotein OprI	12	outer membrane	8835.00	oprI	outer membrane
Outer membrane protein OprG	9		25194.00	oprG	outer membrane
Outer membrane protein OprF	9	calcium ion binding/ porin activity	37640.00	oprF	outer membrane
Outer membrane porin OprE	6		49672.60	oprE	outer membrane
OprD family porin	25		47701.83	oprD_6	outer membrane
OmpA family protein	4		37644.75	IPC1335_18465	outer membrane
Porin	2		37639.00	IPC133_13840	outer membrane
Putative secreted protein	1		8835.00	8835	outer membrane
Cypermethrin hydrolyzing aminopeptidase	1	metalloexopeptidase activity	57561.00	pseA	extracellular
Aminopeptidase	7	metalloexopeptidase activity	57500.57	lieA , lap	extracellular
M28 family peptidase	15	metalloexopeptidase activity	57521.13	GNQ20_03710	extracellular
Putative aminopeptidase	3		57487.33	BN889_03283	extracellular
Keratinase KP1 (Fragment)	1	metalloexopeptidase activity	56937.00	287	extracellular
50S ribosomal protein L17	11	structural constituent of ribosome	14434.55	rplQ	cytoplasmic
6 7-dimethyl-8-ribityllumazine synthase	12	6,7-dimethyl-8-ribityllumazine synthase activity	16231.67	ribH , ribE	cytoplasmic
Chaperonin	1		57086.00	Q041_05796	cytoplasmic
Molecular chaperone GroEL	1		57118.00	IPC123_18905	cytoplasmic
60 kDa chaperonin (Fragment)	1	isomerase activity	56578.00	groL	cytoplasmic
Chaperonin GroEL	12	ATP-dependent protein folding chaperone/isomerase activity/unfolded protein binding	57104.92	groEL	cytoplasmic

Table S5. Subcellular localization of *P. aeruginosa* OMVs

	PA 022 Only		PA ATCC 27853 Only		Overlap	
	Protein types	No.	Protein types	No	Protein types	No.
cytoplasmic	5	46	6	51	6	38
cytoplasmic membrane	4	66	20	218	0	0
periplasmic	4	18	0	0	0	0
outer membrane	15	135	14	78	11	73
extracellular	6	80	1	2	5	27
unknown	15	117	2	28	4	23

Table S6. Clusters of Orthologous Group of *P. aeruginosa* OMV proteins

	PA022	ATCC
cell cycle control, cell division, chromosome partitioning	1	3
defense mechanisms	5	46
cell wall/membrane/envelope biogenesis	140	65
cell motility	33	0
intracellular trafficking, secretion, and vesicular transport	31	50
posttranslational modification, protein turnover, chaperones	30	7
mobilome: prophages, transposons	32	3
energy production and conversion	64	44
amino acid transport and metabolism	0	24
lipid transport and metabolism	9	6
inorganic ion transport and metabolism	26	61
general function prediction only	0	1
function unknown	75	27
translation, ribosomal structure and biogenesis	16	40
